# The Effect of Voluntary Physical Activity in an Enriched Environment and Combined Exercise Training on the Satellite Cell Pool in Developing Rats

**DOI:** 10.3389/fphys.2022.899234

**Published:** 2022-05-25

**Authors:** Samira Rostami, Reyhaneh Salehizadeh, Sahar Shamloo, Rana Fayazmilani

**Affiliations:** Department of Biological Sciences in Sport, Faculty of Sport Sciences and Health, Shahid Beheshti University, Tehran, Iran

**Keywords:** voluntary physical activity, MRF, pax7, pre-puberty, combined training

## Abstract

**Aim:** Postnatal skeletal muscle growth is strongly associated with a satellite cell pool. Early adolescence might be a crucial period when different exercise training interventions have specific consequence on satellite cells. Pax7 and MyoD have been suggested as the leading indicators of satellite cell activation.

**Methods:** In this study, pre-adolescent male rats (*n* = 18) were either subjected to an enriched environment that facilitated physical activities or combined training or control for three weeks. The flexor hallucis longus muscle was removed for biochemical and histochemical analysis.

**Results:** Findings demonstrated that exercise trained rats displayed high levels of serum IGF-1 (*p* <0.05). There was an increase in Pax7 (*p* <0.05) and MyoD (*p* <0.001) mRNA expression. A significant increase in the mean fiber area (*p* <0.01), satellite cell (*p* <0.001), and myonuclear numbers (*p* <0.01) were also observed in both intervention groups. Importantly, enriched rats showed lower corticosterone levels (*p* <0.05) compared to training ones. Regarding performance, trained and enriched rats had significant improvement in forelimb grip strength (*p* <0.01) and load-carrying capacity (*p* <0.05).

**Conclusion:** Type of physical exercise is an essential part in changing satellite cells pool. Different and frequent physical activities in an enriched environment can be effective for muscle development.

## 1 Introduction

Pre-puberty is a critical time for skeletal muscle development. The postnatal myofiber growth seems to be strongly associated with the satellite cells (SCs) pool (Bachman et al., 2018). These types of myogenic cells are located between the basal lamina and sarcolemma of muscle fibers. The proper operation of SCs depends on paired box transcription factor 7 (Pax7) (von Maltzahn et al., 2013). In this case, skeletal muscle growth is controlled by muscle regulatory factors (MRFs), an essential family with important factors (MyoD, Myf5, Myogenin, and MRF4). MyoD expression is known to increase in the time of activation and proliferation of SCs. In particular, co-expression of Pax7 and MyoD has been introduced as the primary indicator of SC activation (Martin and Lewis, 2012). Great SC activity has been observed until the onset of puberty and adolescence (Bachman et al., 2018). In rodents, this period begins at post-natal day (PND) 21 days and lasts approximately 30–49 days of life which is equivalent to 2–3 years old to 12–14 years old in humans (Holder and Blaustein, 2014).

Sensitive postnatal periods are characterized by rapid progress of neuromuscular and motor development (Beunen and Thomis, 2000). In this way, a dramatic increase in muscle fiber area and myonuclear capacity is evident (Bachman et al., 2018). There is abundant evidence that insulin-like growth factor-1 (IGF) is mainly responsible for skeletal muscle development and contributes to SC proliferation and differentiation (Mourkioti and Rosenthal, 2005). Thus, immature myofibers may be more susceptible to stimuli like exercise (Bachman et al., 2018).

The potential role of exercise in SC activation is well clarified (Dhawan and Rando, 2005). Training intervention-induced benefits on muscle structure have been indicated in animal and human studies (Martin and Lewis, 2012). One study specifically described that physical exercise training in 4-week-old rats enhanced SC number and myonuclear content in adulthood. Indeed, exercise exposure during the critical period of growth may have a synergistic role in the SC pool, which lasts into adulthood and leads to sustained effects (Smith and Merry, 2012). The impact of interventions depends on essential aspects of exercise, including the duration, intensity, and type (Martin and Lewis, 2012).

There are various types of physical activities in rodent models, including forced exercise training and voluntary physical activity (Gomes da Silva and Arida, 2015). In this case, endurance and resistance training enhances the SC pool (Martin and Lewis, 2012). However, few works have focused on combined endurance and resistance training during pre-pubertal and juvenile periods. Although the exact mechanisms of SC augmentation have not yet been accurately evaluated, the suitable combination of endurance and resistance training may lead to a more potent stimulus for muscle growth compared to each intervention alone (Murach and Bagley, 2016). Combined training can induce sufficient intensity and lead to changes in SCs by recruitment of fast-twitch fibers (Verney et al., 2008). In addition, it has been demonstrated that concurrent resistance and aerobic training improved muscular fitness (Alves et al., 2016). The relationships between improved muscular performance and IGF-1 have also been described (Cetinkaya et al., 2013), notably because the IGF contribution in SC proliferation and hypertrophy has been demonstrated (Lee et al., 2004). Moreover, muscle hypertrophy has been more evident in resistance than endurance training (Legerlotz et al., 2008). For example, the effects of a resistance training program has been evaluated in adult rats, where increased myogenin, MyoD, and IGF-I mRNA levels with enhanced muscle size was found (Aguiar et al., 2013). However, forced exercise training-induced negative stress could lead to muscle atrophy (Eldomiaty et al., 2020).

Voluntarily physical activities may not induce potential negative stress during forced exercise training (De Bono et al., 2006). In loaded and unloaded conditions, voluntary exercise paradigms have been used in existing animal studies (Smith and Merry, 2012). For example, pre-adolescents rats exposed to resistance running wheels and free-spinning running wheels displayed hypertrophy in different muscles and a significant number of SCs (Legerlotz et al., 2008). Physical enrichment is a novel approach where animals usually have access to running wheels and other motor elements, known as critical parts of enriched environments. In particular, climbing is a natural behavior in young animals. In this regard, resistance activities can be performed through ladders and steps in the cage (Spangenberg et al., 2005). Furthermore, toys and different objects are provided in ample space like a playroom (Sale et al., 2014). The nature of these environmental housing conditions can affect muscle biology by reducing stress levels. Numerous investigators have demonstrated the neural consequences of an enriched environment, but there is less information on muscular adaptations. Recently, one study demonstrated that different enriched environments could increase muscle development (Sudo and Ando, 2021). This finding is crucial because many benefits of enriched environment (EE) are gained in the early periods of life (Sampedro-Piquero and Begega, 2017). However, voluntary activities in an enriched environment are not the same as exercise training programs and may not elicit sufficient muscle load to cause adaptations in SCs and myonuclear domain (Kurosaka et al., 2012).

The satellite cell pool may be affected by the fibers type composition. Although limited information is currently available, there is evidence of differences in the number of satellite cells between different muscles and fibers. In this regard, more satellite cells have been reported in slow muscle and fibers (Yin et al., 2013). On the other hand, it has been found that MyoD expression levels are higher in fast muscles like FHL (Flexor Hallucis Longus) which needs further investigation (Hughes et al., 1997).

The effects of exercise on SCs have been studied, but essential changes during the pre-pubertal and juvenile periods are unclear. This stage of life represents a unique period in skeletal muscle development (Gabel et al., 2017). Exercise interventions may profoundly affect the SC pool and have long-term and enduring consequences. The type of physical training used during this period is an essential issue. The differences between training modalities may result in specific adaptations. Hence, the aim of this study was to assay the effects of voluntary physical activity in an enriched environment versus forced combined training on the SC pool in pre-adolescent rats’ FHL muscles.

## 2 Materials and Methods

### 2.1 Animals

We followed the “guidelines for planning animal research and testing” (Smith et al., 2017). The experimental protocols were approved by the Animal Care Committee of the Shahid Beheshti University (IR.SBU.REC.1398.007). Eighteen pre-adolescent male Wistar rat pups (from Laboratory Animals of the Razi Vaccine and Serum Research Institute, Iran), 15 days old, were maintained in a controlled environment (21–23°C, 56% humidity, and 12-h light-dark reverse cycle) with their mothers. The animals were randomly divided into three groups: enriched environment (EE), combined exercise training (CET), and control group (C) (*n* = 6 per group), following weaning at PND 22 (Rostami et al., 2021a). The subjects were weighed weekly and had ad libitum access to food and water.

### 2.2 Forced Exercise Training and Enriched Environment

The rats in the CET group were subject to endurance) training even days) and resistance training (odd days) during 28–48 PND (Rostami et al., 2021a). First, the rats were adapted to treadmill running (Tajhiz Gostar Company Ltd., 2021) and ladder climbing (100 cm high, 2 cm grid steps, and 80° incline) for three days. Subsequently, the load-carrying capacity of animals was assessed at PND 25 and 26. The animals performed endurance training at 70% of maximum running speed (14–16 m/min). The first training session started with running for 20 min a day and then the duration of training was gradually enhanced to reach 45 min at the end of the third week. Resistance training with eight sets was performed. In this way, the rats climbed the ladder with 50, 75, 90, and 100% from the previous maximal load. When the task was completed, a load of 7 g was added until failure (Hornberger and Farrar, 2004; Huang et al., 2006).

In order to create voluntary physical activity, rats in the EE group were group-housed in large enriched cages (40 × 60 × 90 cm). This environment was composed of three floors, which animals could move among the floors by stairs and climb the cage walls. In addition, there were wheel running and ladders to promote physical activity. The animals had access to food pellets and water ad libitum on each floor and were not forced to do physical activities (Sale et al., 2014).

### 2.3 Physical Performance Assessments

To measure maximal load-carrying capacity, the rats climbed the ladder with a load equal to 75% of their body weight. Then, a weight of 7 g was added to the carrying bag. The maximum strength was calculated when animals could complete the climb (Hornberger and Farrar, 2004). Grip strength was assessed to evaluate the forelimb strength of rats. While the animal tightly grasped the horizontal bar with both paws, their tail was pulled back slowly and at a constant speed. Measurements were recorded when paws released from the bar (Meyer et al., 1979).

### 2.4 Samples Collection

After interventions, animals were anesthetized with carbon dioxide at PND 54. Blood samples centrifuged at 3000 rpm for 10 min at 4°C to extract serum (Eppendorf Centrifuge, 5415R). Next, muscle tissues were removed. Some of the collected FHL muscles (left side) were frozen in liquid nitrogen and keep at 80°C for biochemical measurements. For histological analysis, the rest of the tissues (right side) were fixed in 10% formalin in phosphate buffer. Subsequently, transverse sections with a thickness of 7 μm were prepared using a microtome (Leica, Wetzlar, Germany) at −20 °C.

#### 2.4.1 Enzyme-Linked Immunosorbent Assay (ELISA)

The enzyme-linked immunosorbent assay (ELISA) with 96-well kits (Korain Biotech Co.) was performed to assay basal serum IGF-I (E0709Ra) and corticosterone (E0496Ra) levels in sensitivity of 0.24 and 1.55 ng/ml, respectively (Rostami et al., 2021a). The manufacturer’s instructions were used, and absorption was measured at a 450-nm wavelength.

#### 2.4.2 Gene Expression Analysis (Real-Time Quantitative PCR)

One ml of QIAzol Lysis Reagent (QIAGEN, United States, Cat. No: 79306) was added to 100 mg of tissue and incubated at room temperature for five minutes to extract RNA from homogenized tissues (FHL muscles). Cold chloroform was then added, and the supernatant was transferred to a microtube containing RNAase-free water after centrifugation. Finally, the concentration and purity of obtained RNA were measured by a Nanodrop spectrophotometer (Thermo Scientific, United States). Optical density (OD) was evaluated at 260 and 280 nm, and OD260/280 ratio (around 2.0) indicated the RNA purity. cDNA was synthesized from 1 μg of total RNA. The process of cDNA synthesis was accordance with manufacturer’s guidelines (Thermo Scientific, United States). For this purpose, 10 μL of Dnase-treated RNA was poured into the microtube, and 10 μL of cDNA synthesis kit was added. Finally, real-time PCR was performed by SYBR Premix Ex TaqTM II (Amplicon, Denmark) with an ABI StepOnePlus Real-Time PCR System (ABI Stepone, United States). mRNA level was normalized to the GAPDH mRNA level using the 2^−ΔΔCT^ method. The characteristics of the primers used are presented in Table 1.

**TABLE 1:** The characteristics of the used primers.

Genes	Genbank accession No	Sequence (5′–3′)
*Pax7*	NM_001191984.1	F *CAT​TCT​CAG​CAA​CCC​GAG​TG*
		R *GAGATGGAGGAGGCAGAG*
*MyoD*	NM_176079.2	F *GAC​GGC​TCT​CTC​TGC​TCC​TT*
		R *GTC​TGA​GTC​GCC​GCT​GTA​G*
*GAPDH*	NM_017008.4	F *AGG​TCG​GTG​TGA​ACG​GAT​TTG*
		R *TGT​AGA​CCA​TGT​AGT​TGA​GGT​CA*

#### 2.4.3 Immunohistochemical Analysis

For immunofluorescence staining, sections were washed in phosphate-buffered saline for 20 min (PBST, pH 7.4) and fixed with 4% paraformaldehyde for 15 min, followed by blocking step with 5% normal goat serum (NGS) in PBST for 30 min. After blocking, the muscle sections were incubated overnight with primary antibodies (anti-Pax7 at 1:500; Santa Cruz Biotechnology) in 1% NGS in PBST. After washing the sections, they were incubated for one hour at room temperature (anti-mouse IgG antibody at 1:500; Santa Cruz Biotechnology). Following additional washes, nuclei staining was developed with 4’, 6-diamidino-2-phenylindole (DAPI; 1: 10,000; Sigma Aldrich) and then mounted (Vector Labs, Burlingame, CA, United States).

After the staining procedure, the images were analyzed using a fluorescence microscope (×20 objective) equipped with a digital camera (Olympus, Japan) to detect the satellite cells. The Pax7 (green) and DAPI staining (blue) were visible using the B2A and UV-2E/CT filters, respectively. As described previously, the numbers of SC and myonuclei per muscle fiber were calculated. In total, 15 areas in each muscle cross-section were randomly selected for SCs and myonuclei measurements. In this case, the number of SCs and myonuclei relative to the number of fibers evaluated in each section was determined. The percentage was calculated as the quotient of SCs and myonuclei. Total area was divided by the total number of fibers to determine the mean cross-sectional fiber area (IM 500, Leica) (Kojima et al., 2007).

### 2.5 Statistical Analysis

SPSS 20 Software was applied to analyze the data. Accordingly, normal distribution was first evaluated with the Kolmogorov-Smirnov test. Significant differences between groups were determined by Ordinary one-way analysis of variance (ANOVA) and Tukey post hoc test (*p* < 0.05).

## 3 Results

### 3.1 Body Weight and Performance Measurements

We did not detect notable differences in body weight among groups over the 3 wk of intervention [F (2, 6) = 0.002, *p* = 0.99; Table 2] and at the end [F (2, 15) = 0.28, *p*˃ 0.05]. However, there were remarkable differences in load-carrying capacity [F (2, 15) = 4.27, *p* = 0.03] and compared to C group, 18 and 38% increase were found in the EE and CET groups, respectively. We also observed considerable differences in grip strength [F (2, 15) = 8.20, *p* = 0.003] between groups, such that 19% increase in the enriched rats and 17.7% increase in the trained rats were observed compared with controls. Load-carrying capacity and grip strength did not differ between CET and EE groups.

**TABLE 2:** Bodyweight and performance measurements.

	Body weight (g)	Body weight changes (g)	Maximum weight carrying (g)	Grip strength (g)
C	146/9 ± 3.22	83.18 ± 20.28	111.3 ± 7.79	435.6 ± 16.34
EE	149.2 ± 2.87	81.24 ± 18.45	132.1 ± 11.78^*^	518.6 ± 15.95^**^
CET	147.0 ± 2.81	82.19 ± 18.56	153.6 ± 12.80^*^	512.9 ± 17.69^**^

Data are expressed as mean ± SEM. C, control; EE, enriched environment; CET, combined exercise training.

### 3.2 Serum IGF-1 and Corticosterone Levels

**FIGURE 1:**
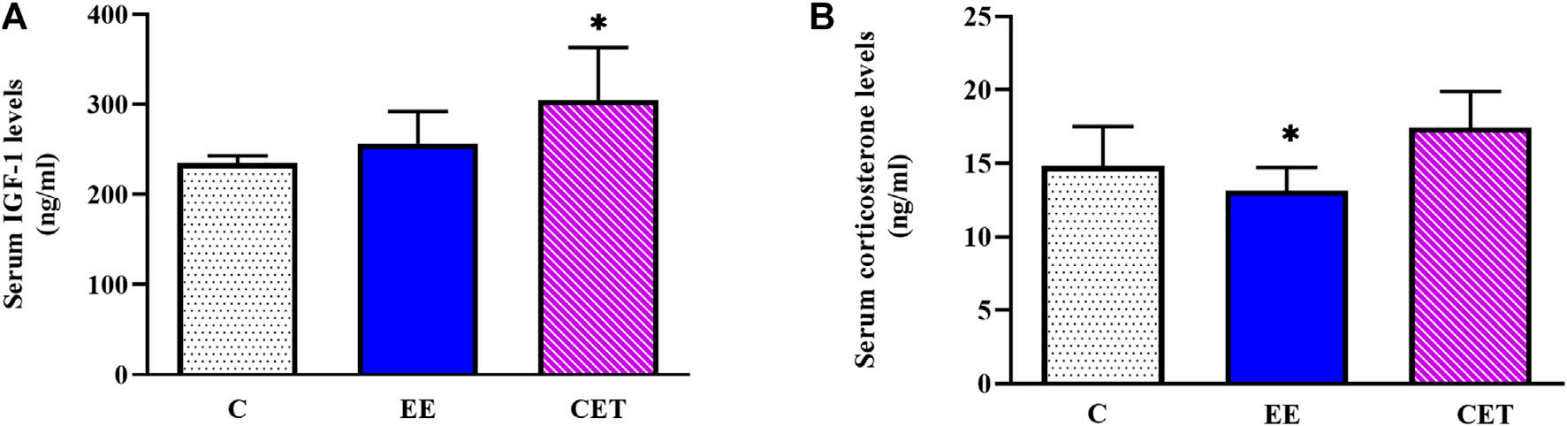
Serum IGF-1 **(A)** and corticosterone **(B)** levels display a significant difference in preadolescent rats subjected to an enriched environment (EE) and combined exercise training (CET). ∗*p* < 0.05, vs. control group.

Serum IGF-1 levels were different considerably between groups [F (2, 15) = 4.78, *p* = 0.03; Figure 1A]. Greater IGF-1 was detected after CET compared with controls (*p* < 0.05), but no significant increase was found in the EE group. IGF-1 did not differ between CET and EE groups. This study also found remarkable differences in the circulating corticosterone levels [F (2, 15) = 5.39, *p* = 0.01; Figure 1B]. The EE rats exhibited significantly lower corticosterone levels compared with CET (*p* < 0.05). No differences were observed between the EE and C groups.

### 3.3 Pax7 and MyoD mRNA Expression

**FIGURE 2:**
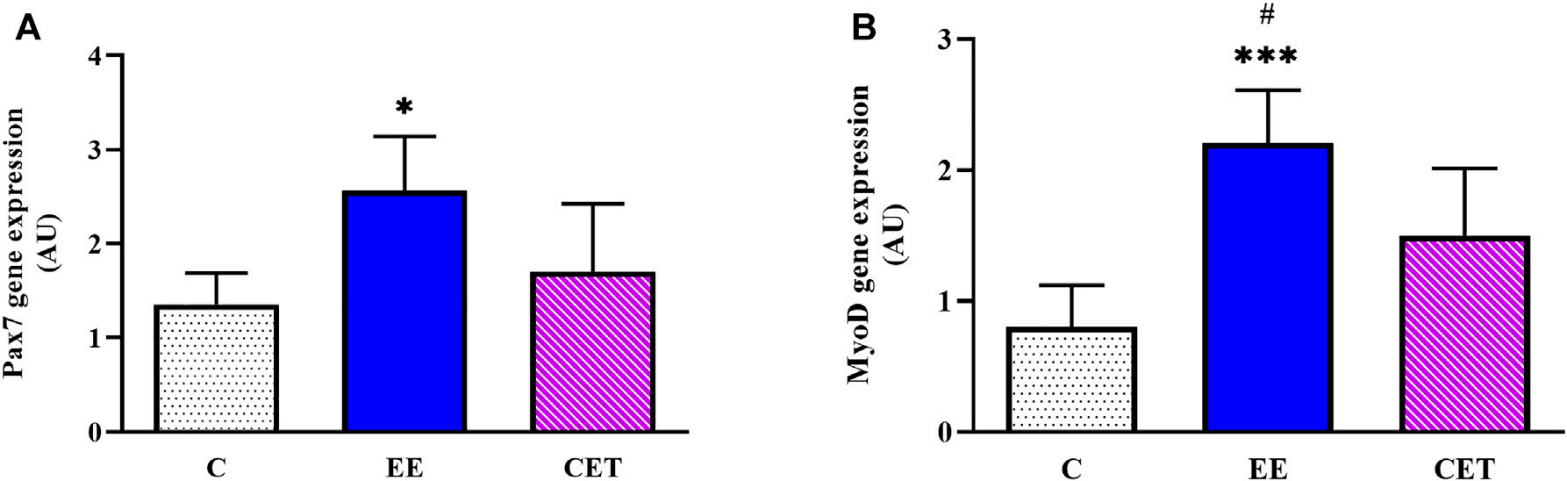
Enriched environment (EE) and combined exercise (CET) significantly increased Pax7 **(A)** and MyoD mRNA levels **(B)** in preadolescent rats. *p < 0.05, ***p < 0.001 vs. control group. #*p* < 0.05 vs. CET group.

Regarding gene expression, there was a remarkable difference between groups in Pax7 levels [F (2, 15) = 6.10, *p* = 0.01; Figure 2A], with a significant increase observed in the EE group compared with C and CET (*p* < 0.05). In addition, greater Pax7 mRNA was found in CET rats than C group, while the changes were not significant (*p* < 0.10). MyoD levels were also different between groups [F (2, 15) = 14.18, *p* = 0.0007; Figure 2B]. The EE group showed significantly greater expression of MyoD mRNA compared to C (*p* < 0.001) and CET (*p* < 0.05) groups. MyoD expression in the CET group was also higher than the C group.

### 3.4 Histological Properties of the FHL Muscle

**FIGURE 3:**
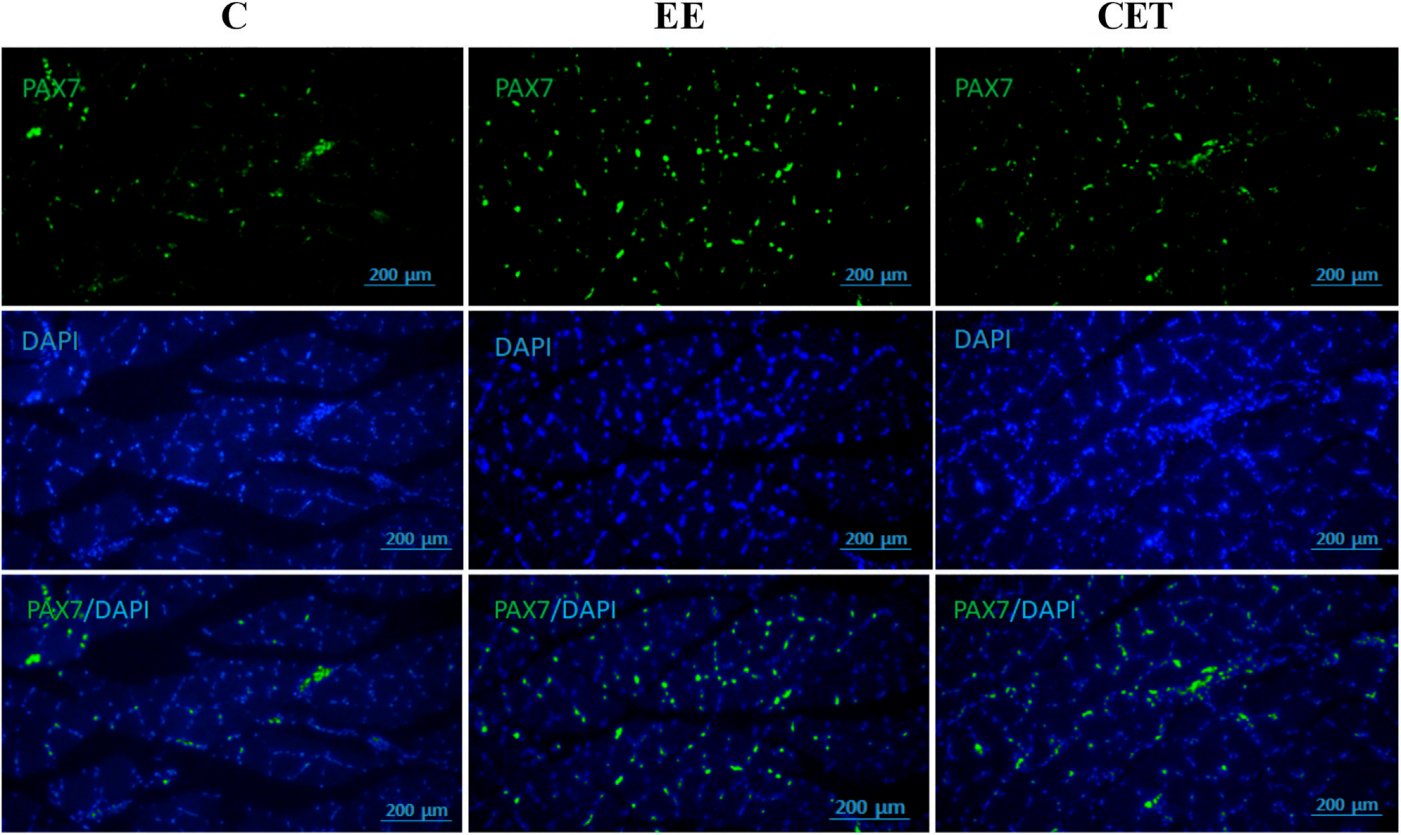
Representative images of satellite cells and myonuclei. The immunostaining staining was used to detect Pax7 and myonuclei.

**FIGURE 4:**
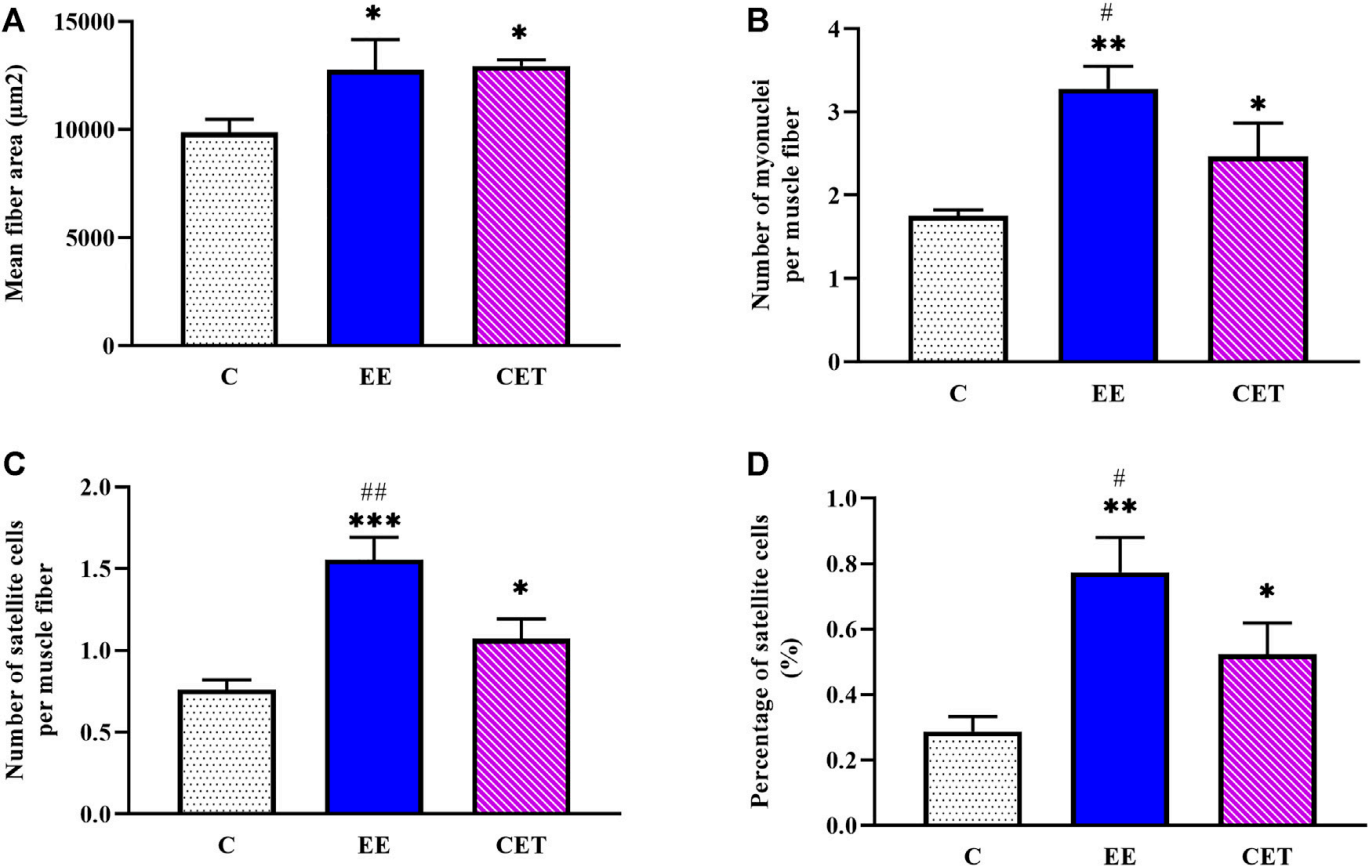
Characteristics of satellite cells and myonuclei in preadolescent rats subjected to an enriched environment (EE) and combined exercise training (CET). A significant increase in mean fiber area **(A)**, the number of myonuclei per muscle fiber **(B)**, the number of SCs per muscle fiber **(C)**, and the percentage of SCs **(D)** was detected. *p < 0.05, **p < 0.01, ***p < 0.001 vs. control group. #*p* < 0.05, ##*p* < 0.01 vs. CET group.

Images of fiber area, satellite cell, and myonuclei by group are presented in Figure 3. Both interventions significantly affected mean muscle fiber area [F (2, 15) = 11.08, *p* = 0.0097; Figure 4A]. Compared to the C group, both EE and CET groups showed increased fiber area (both *p* < 0.05). Similarly, there was a significant difference between groups in the number of myonuclei per muscle fiber [F (2, 15) = 21.77, *p* = 0018; Figure 4B]. Post hoc analysis showed that the number of myonuclei per muscle fiber were greater in EE and CET rats compared with C (*p* <0.01 and *p* < 0.05, respectively). EE rats also had more myonuclei per muscle fiber compared with CET. There was a significant difference in the number of SC per muscle fiber [F (2, 15) = 39.19, *p* = 0.0004; Figure 4C], with more SC per muscle fiber observed in the EE and CET groups (*p* <0.001 and *p* < 0.05, respectively). Moreover, the EE group had significantly more SC than the CET group (*p* <0.05). The percentage of SC also differed between groups [F (2, 15) = 23.59, *p* = 0.0014; Figure 4D). The percentage of SC in EE and CET groups was significantly higher than in the C group (*p* <0.01 and *p* <0.05, respectively).

## 4 Discussion

The present study investigated the effect of two types of training interventions on satellite cells in the pre-pubertal period. This study demonstrated that voluntary physical activity in an enriched environment enhanced the gene expression of Pax7 and MyoD. However, there was a marginal increasing trend in combined exercise training group. Further, both conditions were characterized by significantly greater number of SCs, myonuclei, and mean fiber area of the FHL muscle compared with controls. Notably, the current study found significant differences in enriched rats compared to the training group and remarkably lower serum corticosterone level of the EE group. However, increased serum IGF-1 level was observed only in the combined exercise training. From a physical performance view, three weeks of either intervention improved muscle strength (forelimb grip strength and load-carrying capacity).

Different aspects of exercise can play a role in muscular adaptations (Martin and Lewis, 2012). Some studies have pointed out that running distance in endurance protocols correlated with a large SC pool (Kurosaka et al., 2009). In some studies, long-distance running is also associated with low body weight (Kurosaka et al., 2009). There was no significant difference in animal bodyweight that may be related to the short duration of the current intervention. Lack of remarkable changes in body weight can also be associated with increased lean muscle mass and decreased body fat following exercise protocols. SC activation has been observed in animal models following 6–13 week periods of endurance training (Kurosaka et al., 2009; Kurosaka et al., 2012; Mustofa et al., 2018). Besides, exercise intensity may also be a critical parameter when evaluating the training effects. For instance, SC pool enhancement in adult female rats was reported only following high-intensity running on the treadmill and remained unchanged in high-duration groups (Kurosaka et al., 2012). The trained rats ran on the treadmill at moderate intensity to endurance training. Additionally, progressive resistance training on a vertical ladder was performed three days a week. Regarding the type of training, the effect of endurance (voluntary free wheel exercise) and resistance (resistance wheel exercise) training in pre-adolescent rats was investigated in which enhanced SC numbers were found in both types of exercise (Smith and Merry, 2012). Therefore, it may be speculated that type of physical exercise training may have been a determining factor in the result observed. However, resistance training protocols have mainly reported exercise-induced muscle hypertrophy (Legerlotz et al., 2008). The current findings demonstrated that combined training enhanced Pax7 and MyoD levels. Although gene expression did not differ, trained rats displayed significant differences in the SC number, accompanied by greater myonuclear content and muscle fiber area compared with control rats. In line with these observations, increased SC and MRF expression number has been shown after resistance training in adult rats (Aguiar et al., 2013; Lim et al., 2018). The effects of forced, combined training have been rarely described in juveniles, whereas voluntary exercise models in loaded and unloaded conditions have been used in the pre-pubertal period.

In one study, four-week-old rats were housed with a free-spinning wheel from four to seven weeks of age, and no significant differences in muscle fiber nuclei and SC number were reported (Smith and Merry, 2012). On the contrary, this current study’s intervention increased the number of SCs over three weeks. We provided an enriched environment with several accessories for voluntary physical activity. Since enriched rats had access to running wheels, part of the increase in the SC number could have been due to the high-speed intermittent activity on wheels, as observed by others (De Bono et al., 2006; Legerlotz et al., 2008). Voluntary physical activity and play in a large environment may have contributed to this process (Sale et al., 2014). Furthermore, ladders and stairs provided opportunities for juvenile rats to exhibit climbing behavior in their home cage. The cage walls also enabled rats to use the vertical space to climb (Baumans and Van Loo, 2013). Numerous climbing movements plus voluntary activity in running wheels probably influenced the myonuclear capacity resulting in increased muscle fiber area and MyoD expression (Smith et al., 2001). Following this idea, more myonuclear and large fiber sizes were reported in pre-adolescents rats exposed to resistance wheel training (Smith and Merry, 2012).

Interestingly, we found that enriched rats demonstrated more SCs and myonuclei numbers than the CET group. This group was subjected to cage motor stimuli continuously throughout the intervention. The volume of physical activities in a large environment might be effective in SCs activation and observed differences (Sudo and Ando, 2021). Moreover, there were lower corticosterone levels in the EE group compared to the CET. The duration and intensity of exercise training play role in physiological changes. In this study, according to the existing literature, a moderate-intensity training protocol was used, which may have led to physiological adaptation in the HPA (Hypothalamic Pituitary Adrenal) axis. As the training protocol is probably associated with the initial high activity of the corticosterone response, followed by a gradual adaptation to physical exercise (Leasure and Jones, 2008). On the other hand, social interactions, large spaces for voluntary physical activities, and enjoyment may have reduced HPA axis activity and corticosterone levels in enriched rats (Sale et al., 2014). Enriched animals probably did not experience potential negative stress during forced exercise training (De Bono et al., 2006; Baumans and Van Loo, 2013). Hence, these beneficial aspects of voluntary physical activity in an enriched environment could have led to significantly greater Pax7 and MyoD levels that together have been proposed as a critical indicator of SC activation (Martin and Lewis, 2012).

One possible explanation for a greater number of SC and mean fiber area in trained rats could be the pattern of muscle fibers recruitment given the nature of the physical exercise. Type II fibers are mainly recruited by resistance and high-intensity endurance training. The current study shows that FHL muscle fibers with glycolytic properties were stimulated by resistance activities in both intervention groups (Verney et al., 2008). Consequently, at least part of the increase in the MyoD gene expression may be due to muscle hypertrophy, as evidenced in adolescent rats (Ochi et al., 2011) and positive correlations between MRF expression and muscle hypertrophy (Aguiar et al., 2013). Moreover, increases in myofiber size have been associated with high myonuclear numbers in mouse skeletal muscle during 4–6 weeks of the postnatal period (Bachman et al., 2018). On the other hand, increases in myonuclear number may be related to changes in fiber type composition (Van Wessel et al., 2010). In particular, it has been evidenced that MyoD expression levels are higher in fast muscles (Hughes et al., 1997). In the present research, combining endurance and resistance training (physical activities in an enriched environment) probably plays a role in shifting myofibers toward the center of the muscle spectrum and increasing oxidative-glycogenic fibers. However, this research did not determine muscle fibers type and just studied FHL, often known as the fast-twitch muscle. Fiber type composition may change the satellite cells pool. Further studies are needed to investigate other muscle fiber types.

The number of SCs may also be affected by exercise-induced muscle damage in which damaged fibers release cytokines and growth factors (Carosio et al., 2011). In this case, IGF-1 is well known to increase following resistance training, contributing to muscle hypertrophy. Moreover, muscle-derived IGF-1 has been identified as the primary source of circulating this factor (Chargé and Rudnicki, 2004). The present study revealed that three weeks of combined exercise training increased the serum IGF-1 levels. Mechanical load seems to play a critical role in IGF-1 production (de Alcantara Borba et al., 2020). Regular exercise training, especially ladder climbing, could elicit muscle hypertrophy by stimulating IGF-1 secretion (Lee et al., 2004). On the other hand, IGF-1 level was significantly correlated with improved physical fitness (Cetinkaya et al., 2013). Accordingly, this study found a remarkable increase in load-carrying capacity and grip strength, which could clarify the role of IGF-1 in increasing the observed muscle adaptations. Previous studies have shown that pre-adolescent rats exposed to combined training showed decreased body performance after approximately one month of detraining, consistent with reduced serum IGF I levels (Rostami et al., 2021b). However, the IGF level did not display significant changes in the EE group. In this line, a study reported that long-term wheel running did not influence IGF-1 expression and reduced circulating levels of this factor (Matsakas et al., 2004). Enriched animals were not exposed to regular exercises compared to the training group. The intermittent activities in an enriched environment might not induce sufficient intensity to increase IGF-1 or have led to transient effects (Kurosaka et al., 2012; de Alcantara Borba et al., 2020). Physical activities with high frequency and low-pressure characteristics may activate SCs by affecting extracellular matrix (ECM) components. Therefore, damaged myofibers may release other factors such as hepatocyte growth factor (HGF) and have contributed to increased muscle fiber area and MyoD expression (Kurosaka et al., 2009). It is also imperative to note that increased strength in the pre-pubertal period usually depends on neural adaptations, including increased neuromuscular coordination and the recruitment of more muscle units. From this point of view, appropriate training interventions in this critical period can contribute to developing strength (Myers et al., 2017).

While performance measurements did not show significant differences between the CET and EE groups, higher maximum carrying capacity and lower grip strength were found in trained rats, compared to enriched ones. According to the movement mechanics, it can be realized that climbing a ladder in order to determine the maximum weight carrying is mainly done by involving large muscles and is a kind of gross movement. This function largely depends on the tone and strength of the muscles which are especially enhanced in progressive resistance training. However, grip strength as a fine motor skill requires the use of smaller muscle groups in the hands, wrists, and fingers. This isometric task is performed with strong contractions of the flexors. It seems that the permanent placement of animals in an enriched environment and as a result of numerous climbing movements has been effective in the observed differences (Butterfield et al., 2009; Gentier et al., 2013). Since the mechanisms by which physical exercise affects muscle development during the early adolescent period have been less studied and may be different from those of adulthood, much more work is needed to understand the underlying mechanisms.

In conclusion, combined exercise training or an enriched environment may be effective stimulants for increasing satellite cells and inducing hypertrophy. Type of physical exercise appears to be a critical factor in muscle adaptations. Different and frequent physical activities in an enriched environment with mild stress can be an effective muscle development approach. Moreover, exposure to suitable training interventions during the pre-pubertal period may elicit long-lasting developmental changes, which need to be investigated in future studies.

Practically, physical activity and musculoskeletal pressure are important for children’s development. However, excessive physical stress may limit muscle growth and reduce children’s participation in exercise programs. Since different activities are recommended in childhood, combining enjoyable resistance and endurance activities might be a suitable intervention that contributes to muscle development by stimulating different mechanisms.

## Data Availability

The datasets presented in this study can be found in online repositories. The names of the repository/repositories and accession number(s) can be found in the article/Supplementary Material.
