# Supplementary material for: The Effect of Voluntary Physical Activity in an Enriched Environment and Combined Exercise Training on the Satellite Cell Pool in Developing Rats
Source: Front Physiol. 2022 May 25;13:899234. doi: 10.3389/fphys.2022.899234 (PMC9174454; doi:10.3389/fphys.2022.899234)
Supplement: Supplementary file 1 [file Table1.DOCX]

**Table 1.** The characteristics of the used primers

| **Genes** | **GenBank Accession No** |  | **Sequence (5′ – 3′)** |
| --- | --- | --- | --- |
| Pax7 | NM_001191984.1 | F  R | CATTCTCAGCAACCCGAGTG  GAGATGGAGGAGGCAGAG |
| MyoD | NM_176079.2 | F  R | GACGGCTCTCTCTGCTCCTT  GTCTGAGTCGCCGCTGTAGT |
| GAPDH | NM_017008.4 | F  R | AGGTCGGTGTGAACGGATTTG  TGTAGACCATGTAGTTGAGGTCA |
