# Supplementary material for: The Effect of Voluntary Physical Activity in an Enriched Environment and Combined Exercise Training on the Satellite Cell Pool in Developing Rats
Source: Front Physiol. 2022 May 25;13:899234. doi: 10.3389/fphys.2022.899234 (PMC9174454; doi:10.3389/fphys.2022.899234)
Supplement: Supplementary file 2 [file Table2.DOCX]

|  | **Body Weight**  **(g)** | **Body Weight Changes (g)** | **Maximum Weight Carrying (g)** | **Grip Strength**  **(g)** |
| --- | --- | --- | --- | --- |
| **C** | 146/9 ± 3.22 | 83.18 ± 20.28 | 111.3 ± 7.79 | 435.6 ± 16.34 |
| **EE** | 149.2 ± 2.87 | 81.24 ± 18.45 | 132.1 ± 11.78^*^ | 518.6 ± 15.95^**^ |
| **CET** | 147.0 ± 2.81 | 82.19 ± 18.56 | 153.6 ± 12.80^*^ | 512.9 ± 17.69^**^ |

**Table 2.** Body weight and performance measurements

Data are expressed as mean ± SEM. **C**, control; **EE**, enriched environment; **CET**, combined exercise training.

***P< 0.05, **P< 0.01 vs. control**
