# Supplementary material for: The Effect of Voluntary Physical Activity in an Enriched Environment and Combined Exercise Training on the Satellite Cell Pool in Developing Rats
Source: Front Physiol. 2022 May 25;13:899234. doi: 10.3389/fphys.2022.899234 (PMC9174454; doi:10.3389/fphys.2022.899234)
Supplement: Supplementary file 3 [file DataSheet1.DOCX]

**Data Availability Statement**

Publicly available datasets were analyzed in this study. This data can be found here: https://doi.org/10.6084/m9.figshare.19203905.
